# The Impact of the Affordable Care Act on Dental Care: An Integrative Literature Review

**DOI:** 10.3390/ijerph18157865

**Published:** 2021-07-25

**Authors:** Jihee Song, Jeong Nam Kim, Scott Tomar, Lauren N. Wong

**Affiliations:** 1 Department of Family, Youth, and Community Sciences, University of Florida, Gainesville, FL 32611, USA; 2 Department of Microbiology, College of Natural Science, Pusan National University, Busan 46241, Korea; 3 Division of Prevention and Public Health Sciences, University of Illinois Chicago, Chicago, IL 60612, USA; stomar@uic.edu; 4 School of Special Education, School Psychology, and Early Childhood Studies, University of Florida, Gainesville, FL 32611, USA; laurennwong@ufl.edu

**Keywords:** The Patient Protection and Affordable Care Act (ACA), dental care coverage, access to dental services, national sample, systematic review

## Abstract

The goal of the Patient Protection and Affordable Care Act (ACA) is to increase access to health insurance and decrease health care cost while improving health care quality. With more articles examining the relationship between one of the ACA provisions and dental health outcomes, we systematically reviewed the effect of the ACA on dental care coverage and access to dental services. We searched literature using the National Library of Medicine’s Medline (PubMed) and Thomson Reuters’ Web of Science between January 2010 and November 2020. We identified 33 articles related to dental coverage, and access/utilization of dental care services. This systematic review of studies showed that the ACA resulted in gains in dental coverage for adults and children, whereas results were mixed with dental care access. Overall, we found that the policy led to a decrease in cost barriers, an increase in private dental coverage for young adults, and increased dental care use among low-income childless adults. The implementation of the ACA was not directly associated with dental insurance coverage among people in the U.S. However, results suggest positive spillover effects of the ACA on dental care coverage and utilization by people in the national level dataset.

## 1. Introduction

The Patient Protection and Affordable Care Act (ACA) became law on 23 March 2010. The law included some of the most significant changes to the health care system in U.S. history. The goal of the ACA is to increase peoples’ access to health insurance and decrease health care costs while improving health care quality. The ACA includes many provisions to achieve this goal. Several examples include: insurance providers cannot deny and must provide a plan to individuals who have pre-existing conditions; companies who hire 50 or more full time employees must offer health insurance coverage; and individuals who do not have employer-sponsored insurance must buy health insurance through health insurance in the marketplace or pay a penalty (individual mandate). In addition, young adults were allowed to remain on parents’ health insurance plans until the age of 26 (dependent coverage mandate), federal funding expanded Medicaid coverage (Medicaid expansion), and premium tax credits became available. Furthermore, the ACA required ten essential health benefits that would ensure health plans cover care that patients need. For example, health plans must cover maternity and new born care, mental health and substance use disorder services and preventive and wellness services, and must offer pediatric dental care and vision services [1]. According to the Medical Expenditure Panel Survey, 48.5% of children, 36.0% of working-age adults, and 43.7% of seniors saw a dentist within the preceding 12 months in 2015 [2], and total expenditures for dental services was $135.6 billion in 2018 in the United States [3]. Ultimately, the ACA expands involvement of the federal government in the U.S. health care system [4]. This policy overhaul influenced insurance companies with potential spillover effects on employers [5]. A previous systematic review study reported that several provisions of the ACA led to a decrease in the number of uninsured individuals [6]. Another review on the effect of Medicaid expansion under the ACA reported that Medicaid expansion was associated with recipients’ increased access to healthcare [7]. 

The ACA did not explicitly include dental care coverage, but several provisions with the act might influence underserved populations’ access to dental services in a number of ways. For example, Medicaid expansion and the dependent mandate of children being insured under parents’ coverage until the age of 26 might have positively influenced young adult dependents’ and Medicaid recipients’ dental coverage and utilization [5,8]. The number of studies that examined the relationship between singular ACA provisions and dental health outcomes has increased in recent years. However, the results from these studies have not been synthesized. To examine the effect of the ACA on oral health outcomes, it is important to synthesize published studies that use a range of data sources. To our knowledge, there has been no prior review that comprehensively examined the impact of the ACA on oral health outcomes for people in the United States. 

The purpose of this study was to systematically review the effect of the ACA on dental health outcomes, including dental care coverage and access/utilization of dental services. We also conducted a meta-analysis to evaluate the strength of evidence for the effect of the ACA on dental care utilization. The supplement to this article provides a methodology and results of the meta-analysis. Synthesized evidence from our findings can provide information to policy makers and other stakeholders interested in the ACA’s potential effects on oral health care in the U.S. 

A structured and systematic review on the effect of the ACA on dental coverage, and access/utilization of dental care services was conducted by using the Preferred Reporting Items for Systematic Review and Meta-Analysis (PRISMA) guidelines [9]. The following research questions guided this systematic review: (1) Did the ACA improve dental health care coverage among people in the U.S.; (2) Did the ACA increase peoples’ access to or utilization of dental care services; and (3) Did the ACA lead to decreased emergency department (ED) visits for non-traumatic dental conditions. 

## 2. Materials and Methods

### 2.1. Literature Search 

We searched literature by using the National Library of Medicine’s Medline (PubMed) and Thomson Reuters’ Web of Science that was published between January 2010 and November 2020. We used the keywords “Patient protection and Affordable Care Act” and “dental* or oral*” in MeSH Terms, title, abstracts, or topics/keywords to find relevant studies. The detailed search strategy is presented in Table 1. Based on this search terminology, we identified a total 258 publications including 134 from PubMed and 124 from Web of Science.

### 2.2. Inclusion Criteria and Screening 

The inclusion criteria were based on whether the study examined one or more elements of the ACA’s implementation and dental care coverage or access to, and utilization of, dental services. If the publication studies included any outcomes that related to dental care coverage or utilization, the study was included in this review. Only English language studies were included in this review. We included studies of children or adults. We included studies that compared pre- and post-ACA legislation as well as articles that examined only the post-enactment period. However, studies that described legislative, ethical, legal, or political aspects of the ACA provisions, prediction of the ACA, or only explored data prior to implementation, such as baseline assessment of the ACA. Articles not available in full text were excluded from the review as well. 

### 2.3. Data Extraction 

**Figure 1:**
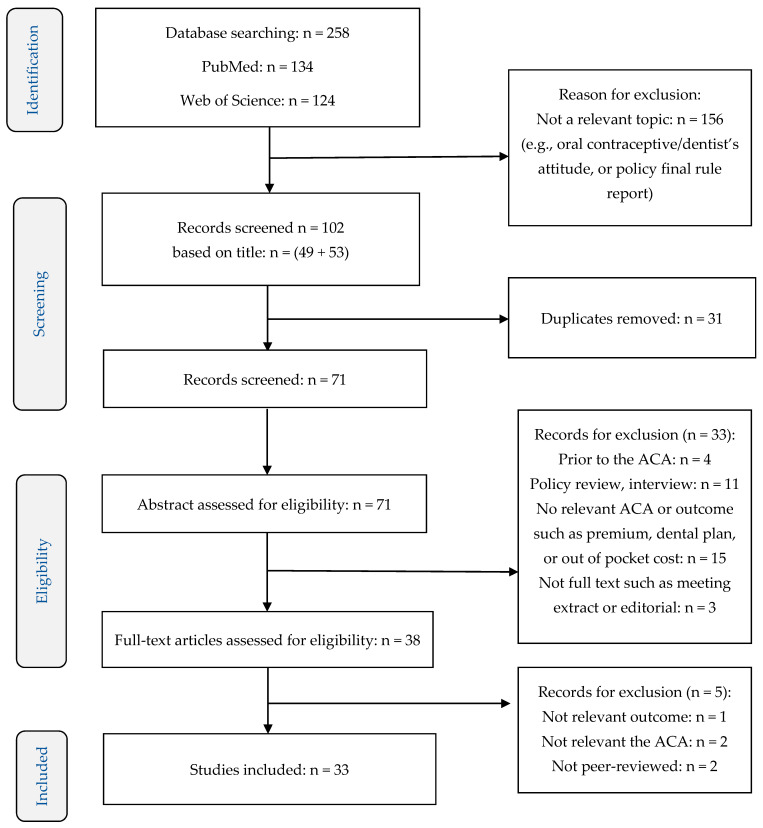
Flow chart of Study Selection.

We obtained 258 records from two web engines by using the specified search terms mentioned earlier. Eligibility of assessment for each included study was performed by two reviewers (JS, JNK) with ongoing discussion about screening and extraction. Each title was examined to exclude irrelevant topics, in which 156 records were eliminated. After excluding duplicate papers, the reviewers examined the abstracts from 71 articles, and then 33 records were eliminated based on the inclusion and exclusion criteria mentioned above. The full text for 38 articles was reviewed and five papers were excluded because they were either not relevant or had not been peer-reviewed. Finally, 33 papers were included for this systematic review. The study selection progress is illustrated in Figure 1.

## 3. Results

### 3.1. Description of Studies

Table 2 lists the final set of 33 papers with brief descriptions of samples, datasets, outcome measurements, and findings. There was more evidence for dental care access (five studies) or utilization (22 studies) than dental care coverage (eight studies) or emergency department (ED) visits (five studies), whereas some studies examined both dental care coverage and utilization. For dental care coverage and utilization, most studies used survey data with a representative sample corresponding with the U.S. population, including the Behavioral Risk Factor Surveillance System (BRFSS), the National Health Interview Survey (NHIS), the Medical Expenditure Panel Survey (MEPS), and the Gallup Healthways Wellbeing Index Survey. For studies examining ED visits for non-traumatic dental conditions, most used the State Emergency Department Database (SEDD), except for one study that used the Nationwide Emergency Department Sample (NEDS). Most studies included samples of adults and only four studies included samples of children. Many studies (15 studies) used a quasi-experimental study design with the difference-in-differences analysis method. Most studies included comparison groups, although the selected years indicating the pre- and post-ACA implementation period varied across studies.

### 3.2. Dental Care Coverage for Adults

There were six publications [5,8,10,11,12,13] that examined the impact of the ACA on dental care coverage among the U.S. adult population. Most studies used the difference-in-differences analysis that compared young adults aged 19–25, 20–24, or 25 years to slightly older adults between periods before and after ACA implementation. One ACA provision allows young adults aged between 19–25 years to remain on their parents’ health insurance policies. The dependent mandate became effective on private health insurance policies on 23 September 2010. All studies showed the increase in private dental benefits after the ACA at varying degrees of magnitude. For example, private dental insurance for the 19–25 year-old group increased by 6.7 percentage points in the post-ACA period (2011) compared with the 27–30 year-old group. Ref. [5] A study using the same data with an extended post-ACA period (2013) reported that private dental coverage for the 25 year age group increased by eight percentage points in post-ACA period compared with the age 27 year group, relative to pre-ACA (*p* < 0.05) [12]. According to a more recent study, the 19–25 year age group had higher rates of private dental coverage compared with the 27–30 year age group after implementation of the dependent mandate [8]. Similarly, dental plan enrollment for the 20–24 years age group increased by an average of 1.3 percentage points, relative to the group aged 30–34 in the post-ACA period (2011–2013) [11]. The largest increase in private dental insurance rates was for young adults aged 19–25 years with a household income in the 125%–400% of the federal poverty level (FPL), whereas there was no significant effect on young adults with a household income less than 125% FPL [5]. There was a significant increase in dental care coverage among young adults aged 19–25 years with low income (<200% FPL) or with higher income (≥200% FPL) compared with adults aged 27–30 years [8]. However, there was no decrease in the dentally uninsured rate in Rhode Island after implementation of the ACA [10]. 

### 3.3. Dental Coverage for Children

There were two studies that examined the impact of the ACA on dental care coverage for children [14,15]. The ACA required insurance plans to include a package of health benefits. Although the purchase of dental insurance was not mandatory, the plans required ten essential benefits including pediatric dental care coverage throughout the embedded benefits in medical health plans or stand-alone dental plans. Private dental coverage for children affected by the ACA’s essential health benefits package increased by 4.6 percentage points as compared with children who were not affected by the ACA’s essential health benefits in the post-ACA policy period (2014–2015) [14]. Another study found that, among children who obtained private medical insurance from health insurance marketplaces in 2014–2015, 23.7% of those living in states where dental coverage was embedded in health plans reportedly had dental care coverage, compared with 14.5% of those in states where purchase of dental coverage was optional, and 9.0% of children in states where dental coverage was required in the marketplace [15]. 

### 3.4. Dental Care Access and Utilization

The majority of studies focused on the effect of Medicaid expansion on dental care access and utilization [8,11,16,17,18,19,20,21,22,23,24,25,26,27,28,29]. Only three studies examined dental care utilization among children [14,23,30]. Several studies used difference-in-differences analysis, but the post-ACA time period and age of the study populations varied by study. 

There was no significant change between 2009 and 2012 for 19 to 25 year-olds compared with 25- to 34-year-olds in the percentage who reported being unable to afford care in the preceding year (DID = −2.6%, 95% CI, −5.61 to 0.61) [20]. The percentage of adults aged 19–25 who reported financial barriers to dental care declined by 2.1% in both 2011 and 2012 compared with adults aged 26–34, relative to the pre-ACA period, but those declines were not statistically significant [13]. In addition, low-income adults who lived in Medicaid expansion states were less likely to get necessary dental care or delaying care than those in the non-expansion states to report being unable, but the difference was not statistically significant (12.5% vs 14.0%, PR = 1.19, CI 0.98–1.43) [18]. However, low-income women who were newly eligible for Medicaid in Ohio had significantly lower odds of reporting an unmet dental care need in 2015 than in 2012 (OR = 0.72, 95% CI 0.54, 0.95) [17]. Adults who were uninsured for some period in the preceding 12 months were much more likely than those with continuous Medicaid coverage throughout that period to report that they delayed receiving or were unable to obtain dental care [24]. 

A number of studies reported increases in rates of dental care utilization and compared those increases between Medicaid expansion and non-expansion states. The rate of dental visits per 1000 persons per month was significantly higher in the post-ACA period (2014–2015) than in the pre-ACA period (2012–2013) [19]. Low-income adults in non-Medicaid expansion states had a mean of 0.08 fewer dental care visits than in Medicaid expansion states [18]. A statistically significant increase in dental care use in 2016 was found among low-income nonelderly adults in Medicaid expansion states with dental benefits than those in non-expansion states with or without adult dental benefits [29]. Wehby et al. [27] reported that low-income adults who lived in Medicaid expansion states that offered extensive dental coverage were 5.8 percentage points more likely to have a dental visit in 2016 when compared to 2012 (*p* < 0.01). Furthermore, Lyu [22] reported a significant increase in the use of preventive dental services among those who were newly eligible for Medicaid and resided in Medicaid expansion states offering extensive dental benefits, compared with those living in non-expansion states that offered extensive dental benefits. 

However, some studies did not find an impact of Medicaid expansion on the use of dental services. There was no significant effect of Medicaid expansion on dental visits for low-income adults [25,31]. Additionally, low-income adults in expansion states with dental benefits had increases in dental care use in 2014 when compared to low-income adults residing in states with or without Medicaid expansion and/or adult dental benefits; however, most of the increases were small and not statistically significant [32]. In addition, the probability of having had a dental visit in the preceding year among low-income residents of Medicaid expansion states that provided dental benefits decreased from 52.3% in 2010 to 50.4% in 2014 (*p* < 0.001) [26]. 

The probability of dental care utilization and the number of dental office visits per month decreased when young adults turned 26 [33]. The rate of receiving an annual dental visit for young adults aged between 18–25 increased from 55.2% in 2009 to 60.9% in 2012 (*p* < 0.001) [21]. Although the degree of difference between years slightly declined when insurance types entered the model (full year private, full year public, partial year uninsured, and full year uninsured), adults aged 18–25 were more likely to have an annual dental visit in 2012 when compared to 2009 (OR = 1.3; 95% CI: 1.1–1.4) [21]. Similarly, oral care use for the 20–24 age group who had employer sponsored dental insurance increased by 0.37 percentage points after implementing the dependent mandate compared with the 30–34 age group [11]. Dental treatment for the 25-year-old group increased by 4.8 percentage points in the post-ACA period compared to the 27-year-old group, relative to the pre-ACA period, but there was no significant effect of the dependent mandate on preventive dental service use [12]. Dental care visits for adults aged 19–25 increased by 2.8 percentage points in 2011 and 3.3 percentage points in 2012 compared with adults aged 26–34, but the increase was only statistically significant in 2012 [13]. 

Several studies reported the effects of the ACA on dental utilization in sub-populations. Medicaid expansion increased the probability of having dental care use for childless adults by 2.5 percentage points [16]. Among the states that provided dental benefits, the probability of having had a dental visit in the preceding year for childless adults residing in expansion states increased from 48.6 percentage points in 2010 to 50.4 percentage points in 2014 (*p* < 0.0001) [26]. Furthermore, Medicaid expansion increased the probability of a dental visit among low-income adults without children by 2.3 percentage points (*p* < 0.05) [34]. These three studies [16,25,34] all used the BRFSS and included samples of low-income adults aged 19–64 years, but the three studies used different post-expansion periods. However, among the states that provided dental benefits, the probability of having had a dental visit for low-income parents residing in expansion states decreased from 56.0% in 2010 to 47.9% in 2014 (*p* < 0.0001) [26]. In addition, there was no statistical difference in dental care use between pre- and post-Medicaid expansion periods among low-income women aged 19–44 [17]. Although children affected by the ACA’s essential health benefits increased their dental care usage, for children not affected by affected by the ACA’s essential health benefit, the increase was not statistically significant [14]. 

### 3.5. Emergency Department (ED) Visits

There were five studies that examined the impact of the ACA on the use of EDs [35,36,37,38,39]. There was an overall increase in ED visits for dental conditions from 2012 to 2014, but there was a decrease in ED visits for dental conditions in states that expanded Medicaid offering dental benefits [38]. There was a 9.7 percentage point decrease in ED visits for non-traumatic dental conditions between 2008 and 2014 in Minnesota [37]. Similarly, the percentage of ED discharges with non-traumatic dental conditions decreased in New York and New Jersey from 2010 to 2014 [39]. The percentage of the ED discharges for dental or oral conditions that occurred among the uninsured decreased from 56.8% in 2013 to 20.5% in 2014 in Kentucky [35] as well as in New Jersey and New York from 2010 to 2014 [39]. However, the percentage of ED discharges for a dental or oral condition that was covered by Medicaid increased in Kentucky (17.6% in 2013 and 49.7% in 2014) [35] as well as in New Jersey [39]. Among Medicaid enrollees, the percentage of those diagnosed with dental or oral health conditions increased from 2.7% in 2013 to 3.7% in 2014 [35].

## 4. Discussion

We systematically reviewed the effect of the ACA on dental coverage and use of dental services. This review of studies conducted so far clearly showed that the ACA resulted in gains in dental care coverage for adults and children, but there were mixed results on its impact on dental care access or utilization. Furthermore, the supplemental meta-analysis we conducted indicated that there was an increase in the use of dental service uses, although it was small and not statistically significant (Figure S1) with evidence of publication bias (Figure S2). Under the ACA, there are several provisions that influence dental care coverage. Individuals are mandated to have health insurance through employers or the marketplace. The ACA allows young adults to remain on their parents’ insurance until age 26 which thus helped to increase dental coverage for the population of young adults aged 18 to 26. In addition, the ten essential health benefits in the ACA required pediatric dental coverage throughout the embedded benefits in medical health plans or stand-alone dental plans. Although Medicaid expansion is optional, all Americans living below 138% of federal poverty level are eligible for Medicaid [26,31], it was estimated that the ACA would make 16 million additional Americans eligible for Medicaid coverage [26,40,41]. While Medicaid provides dental care coverage for children in all states, not all states provide comprehensive dental benefits to adult Medicaid enrollees [42]. As of February 2021, 38 states and the District of Columbia had decided to expand Medicaid [43]. Although the scope of dental coverage varies (extensive, limited, or emergency only benefits), as of September 2019, most states (47) provide some level of dental care coverage for adults enrolled in Medicaid [44].

Previous studies showed that the increase in dental care use was related to dental care coverage [45,46,47]. However, there were mixed effects of the ACA on dental care access and utilization. Most studies did not show an effect of the ACA on increasing access to dental services [13,18,20]. However, in their sensitivity analysis, Kotagal et al. [20] reported that insured people were more likely than uninsured people to be able to afford dental care. For dental care utilization, some studies concluded that the enactment of the ACA led to increased dental service utilization. For example, dental care utilization by low-income nonelderly adults in the Medicaid expansion states with dental benefits increased in 2016 compared to those in non-Medicaid expansion states with or without adult dental benefits [29]. The increase in utilization was reportedly higher in Medicaid expansion states that offered extensive dental coverage [27]. Consistent with those finding, low-income adults in non-Medicaid expansion states had fewer dental care visits than those in Medicaid expansion states [18]. In addition, dental care utilization by young adults aged 18–26 influenced by the dependent mandate provision increased in the post-ACA period when compared with their counterpart age groups [11,12,13,21,48]. However, some studies reported no significant effect of the ACA on dental care utilization. Simon et al. [25] and Kino and Kawachi [49] reported no significant effect of Medicaid expansion on dental visits for low-income adults. Similarly, the probability of having had a dental visit in the past year among low-income residents of Medicaid expansion states with dental benefits significantly decreased from 2010 to 2014 [26]. Furthermore, there was no statistical difference in dental care use between the pre- and post-Medicaid expansion period among low-income women aged 19–44 as well as children affected by the ACA’s essential health benefits [14,17].

Interestingly, Medicaid expansion increased the probability of dental care use among childless adults [16], [25], while, within Medicaid expansion states that provided dental benefits, the probability of low-income parents having had a dental visit decreased from 2010 to 2014 [25]. In addition, the probability of low-income adults, regardless of whether they were parents, residing in Medicaid expansion states having had a dental visit declined in 2014 (the post-ACA period) [26]. This apparent inconsistency might be explained by several possible factors. First, there may be an insufficient supply of dental care providers. The supply side constraints can limit dental care access for new and existing Medicaid enrollees. The likelihood of a dental visit increased in states with a supply of dentists [27]. However, there may not have been a sufficient increase in the supply of dentists to match population growth in some states (e.g., the U.S. average of 58.7 dentists per 100,000 population in 2008 vs 61.0 dentists per 100,000 population in 2020) [50]. Additionally, participation rates of dentists in state Medicaid programs is frequently low because many dentists are reluctant to accept low reimbursement rates, administrative burdens such as paperwork, and changing regulations, or deal with the social stigma of caring for Medicaid patients [51]. Second, other factors such as health literacy might influence dental care use. Health literacy has been found to have a moderating effect on the increase in dental care use during a period of increased dental insurance coverage [49]. Third, the post-ACA covered in recent studies may not have been long enough to capture the long-term effects of the ACA. For example, there are inconsistent results between 2014 and 2016 in Nasseh and Vujicic’s two studies [29], [32] that used different years for the post-ACA period. In addition, people in the U.S. remain on long waiting lists to apply for Medicaid, which may put people into another insurance category [10]. Finally, there were discrepancies between the rates of Medicaid enrollment and dental care use. The number of Medicaid beneficiaries in Rhode Island who received any dental services of at least once a year increased between 2013 and 2015, but the percentage of those who received any dental care services decreased between 2013 to 2015 [28]. 

Regular preventive care can avoid and reduce costs because problems can be diagnosed in an early state and treated before worse symptoms develop. The inability to access regular dental care leads to poor oral health outcomes [52]. Lack of affordability of dental care may lead underserved populations to use local hospital emergency departments (ED) as primary dental care facilities. There was some evidence that the ACA reduced the rate of ED visits for dental problems. 

This systematic review addresses gaps in the existing literature that might be addressed in future studies. Notably, there was a lack of distinction between preventive dental services and restorative or surgical dental services in the existing literature. Furthermore, the dependent coverage mandate has been in effect since 23 September 2010. Most states began Medicaid expansion in January 2014, although some states such as California, Connecticut, The District of Columbia, and Minnesota expanded their Medicaid programs before 2014 [26]. Most investigation of the ACA on dental care used datasets from 2016 or earlier, so most studies reviewed examined the early impact of the ACA. As mentioned above, there were inconsistent results in assessing differences in dental care service utilization between 2014 and 2016. It might be insufficient to capture the effects of the various ACA provisions in their early years of implementation. Thus, further research is required to demonstrate the full effect of the ACA on dental care access and utilization. 

In 2017, Congress passed the Tax Cuts and Jobs Act, effective on 1 January 2019, which eliminated the individual mandate penalty. The Congressional Budget Office estimated that repealing the individual mandate requirement would reduce health insurance enrollment by 3 million to 6 million between 2019 and 2021, with a resulting 10% increase in premiums in the individual market [53]. Those effects might significantly impact dental care coverage and utilization. This change might reduce the increased rates of dental coverage that were highlighted in this review. Future studies would benefit from our knowledge about health care by examining the repeal of the ACA on dental care coverage and use. 

There are several limitations to this systematic review of findings. First, all of the included studies were observational study designs, so there are inherent limitations in being able to draw causal inferences about the impact of the ACA on coverage and utilization. Furthermore, as mentioned above, the ACA did not directly apply to dental health outcomes, thus we should be cautioned to draw the causal inferences between the passing of the ACA and dental health outcomes. However, the purpose of this review was to synthesize the peer-reviewed literature that examined the effect of the ACA on dental health outcome. Second, we did not include gray literature that is not peer-reviewed in our study. That omission may have led to a potential publication bias if positive findings are more likely to be published in peer-reviewed journals than in the gray literature [7,54]. However, most studies we selected used large nationally representative samples’ datasets, so the positive findings were not likely due to publication bias. 

## 5. Conclusions

Assessing the impact of the ACA on dental care use is challenging because the law did not directly apply to dental insurance coverage. However, the ACA supported Medicaid expansion and most states that participated in Medicaid expansion had more of its residents covered for all Medicaid services including dental services [24]. The positive effects of the ACA found in the national level dataset suggest that there were spillover effects of the ACA on dental care coverage and utilization. As mentioned, the ACA does not directly apply to dental care coverage, but the policy led to a decrease in cost barriers, an increase in private dental coverage, and an increase in dental care use among both young adults [11,12,13,21] and low-income childless adults [16,25,26]. 

The findings of the positive impact of the ACA on dental care utilization should be kept in mind as the Biden–Harris administration and health policy makers, such as Congress, the Centers for Medicare and Medicaid Services, and individual states, evaluate new health care plans. Repeal of the ACA would likely have a negative impact on dental care coverage and access for a significant portion of people in the United States.

## Figures and Tables

**Table 1:** Search terms for literature search.

Search Engine	Search Term
PubMed	“Patient protection and Affordable Care Act” [MeSH Terms] ^†^ AND (dental* OR oral*) [Title/Abstract]
Web of Science	“Patient protection and Affordable Care Act” OR “Affordable care act” [Topic] AND (dental* OR oral*) [Topic]

^†^, MeSH: Medical Subject Headings.

**Table 2:** Findings from Studies of effects of Affordable Care Act.

Citation	Data Sources, Analyses	Study Population	Measures	Findings
Shane and Ayyagari (2015) [5] ACA (dependent expansion)	The MEPS, 2006–2009, and 2011 Difference-in-differences analysis	Adults aged 19–25 and 27–30	Private dental coverage	Private dental coverage for adults aged 19–25 increased by 6.7 percent points in 2011 compared to adults aged 27–30, relative to pre-ACA (*p* < 0.05). Private dental coverage for adults aged 19–25 with household incomes between 125 and 400 FPL increased by 14 percent points compared to adults aged 27–30, relative to pre-ACA (*p* < 0.05). There was no significant effect of the dependent mandate on private dental coverage among poorer (<125 FPL) or higher household income (>400 FPL) individuals.
Shane and Wehby (2020) [8] The dependent mandate	The MEPS, 2006–2015 Difference-in-differences	19–25 years and 27–30 years	Private dental insurance Preventive dental service use Dental treatment Any (preventive + treatment)	Regardless of race, young adults (19–25 years) had higher rates of private dental coverage after the implementation of the dependent mandate compared with adults aged 27–30 years (non-Hispanic black [12 percentage points increase]; Hispanic [7 percentage points increase]; and non-Hispanic white [8 percentage points]). Among non-Hispanic blacks aged 19–25 years, dental visits increased by 7.8 percentage points after the implementation of the dependent mandate, compared with non-Hispanic black adults 27–30 years (CI: 0.013–0.143). Among non-Hispanic blacks aged 19–25 years, preventive dental visits increased by 5.6 percentage points after the implementation of the dependent mandate, compared with non-Hispanic black adults aged 27–30 years (CI: −0.001–0.113); however, there was no statistically significant changes in receipt of dental treatment for these age 19 to 25. There were no statistically significant changes in preventive treatment, or both services for non-Hispanic white and Hispanics. There were significant increases in dental coverage among young adults (19–25) with low income (<200% FPL) and with high income (>200% FPL) compared with adults aged 27–30 (7.1 percentage points in low-income adults vs 9.2 percentage points in high-income adults). There were no significant changes in dental service use among low-income adults aged 19–25 compared with low-income adults aged 27–30, whereas there was a marginally significant increase in any dental visits (3.6 percentage points) and dental treatment (2.4 percentage points) among high-income adults aged 19–25 compared with high-income adults aged 27–30.
Dumont, Oh, and Copper (2017) [10]	The BRFSS in Rhode Island, 1 January–1 October, 2013 and full year of 2014	Adults age 18 and older	Absence of dental coverage between 2013 and 2014	The percentage of dentally uninsured adults did not change significantly: 29.6% (95% CI 28.0–31.2) in 2014 compared with 32.5% (95% CI, 30.2–34.7) before October 2013.
Huang (2018) [11] Dependent expansion	Delta Dental of Michigan, 2008–2013 Difference-in-differences linear regression	Young adults aged 20–24 years and older adults 30–34 years	Dental plan enrollment Oral health care use rates	Dental plan enrollment among young adults increased 1.3 percentage points after implementing dependent expansion, relative to the older adult group (*p* < 0.01). Oral care use among young adults increased 0.39 percentage points after implementing dependent expansion, relative to the older adult group (*p* < 0.05).
Shane and Wehby (2017) [12] ACA (dependent mandate)	The MEPS, 2006–2013 Difference-in-differences analysis	Adults aged 25 and 27	Private dental coverage Preventive dental service use Any dental treatment	Private dental coverage for 25 years increased by eight percentage points in post-ACA compared with 27 years, relative to pre-ACA (*p* < 0.05). There was no significant effect of dependent mandate on preventive dental service use among 25 years compared with 27 years. Dental treatments for 25 years increased by 4.8 percentage points in the post-ACA compared to 27 years, relative to the pre-ACA (*p* < 0.05).
Vujicic, Yarbrough, and Nasseh (2014) [13] ACA (dependent expansion)	The NHIS, 2008–2012 Difference-in-differences	Adults aged 19–25 and 26–34	Private dental benefit coverage Dental care visits in the last 12 months Financial barriers to obtaining needed dental care	Private dental benefit coverage for adults aged 19–25 increased by 5.6% in 2011 compared with adults aged 26–34 (*p* < 0.01), relative to pre-ACA passing (2008–2010). Private dental benefit coverage for adults aged 19–25 increased by 6.9% in 2012 compared with adults aged 26–34, relative to pre-ACA (2008–2010) (*p* < 0.01). Dental care visits for adults aged 19–25 increased by 2.8% in 2011 compared with adults aged 26–34, relative to pre-ACA (*p* = 0.062). Dental care visits for adults aged 19–25 increased by 3.3% in 2012 compared with adults aged 26–34, relative to pre-ACA (*p* = 0.038). The percentage of adults aged 19–25 reporting financial barriers to dental care declined by 2.1% in 2011 compared with adults aged 26–34, relative to pre-ACA (*p* = 0.068). The percentage of adults aged 19–25 reporting financial barriers to dental care declined by 2.1% in 2012 compared with adults aged 26–34, relative to pre-ACA (*p* = 0.087).
Kranz and Dick (2018) [14] ACA (10 essential health benefits)	The NHIS, 2010–2015 Difference-in-differences multivariate linear probability regression	Children aged 1–18 without public health insurance (n = 16,404)	Rates of dental insurance Rates of dental visit in the past 12 months	Private dental coverage increased by 4.6 percentage points among children who were affected by the ACA’s essential health benefits (EHB) compared to children who were not affected by the ACA’s EHB in 2014–2015, relative to the pre-ACA period of 2010–2013 (*p* = 0.013). The percentage of having a dental visit in the past year increased by 2.7 percentage points for children who were affected by the ACA’s EHB compared to children who were not affected by the ACA’s EHB in the post ACA period, but it was not statistically significant (*p* = 0.071).
Kranz and Dick (2019) [15] ACA (dental coverage via the market place)	The NHIS, 2014–2015	Children age 0–18 (n = 696)	Parent reported rate of pediatric dental coverage	During 2014–2015, 14.5% of children obtaining medical insurance from marketplaces offering optional dental coverage reported they had dental insurance. During 2014–2015, 9.0% of children obtaining medical insurance from marketplaces requiring purchase of dental coverage reported they had dental insurance. During 2014–2015, 2015, 23.7% of children obtaining medical insurance from marketplaces with dental coverage embedded in all medical plans reported that they had dental insurance.
Cawley, Soni, and Simon (2018) [16] Medicaid expansion	The Behavioral Risk Factor Surveillance System (BRFSS), 2010–2016 Difference-in-differences analysis	Low-income childless adults aged 19–64 (n = 80,200)	A dental care visit in the past 12 months	Medicaid expansion increased the probability of having visited a dentist in the past 12 months by 2.5 percentage points (95% CI 0.0001–0.050, *p* = 0.05).
Farietta, Lu, and Tumin (2018) [17] Medicaid expansion	Data: Ohio Medicaid Assessment Survey (OMAS), 2012 and 2015 Logistic regression	Low-income women aged 19–44 who were newly eligible for Medicaid after expansion (n = 489 in 2012 & 1273 in 2015)	Unmet dental needs during the past 12 months A dental care visit in the past year	Low-income women had significantly lower odds of reporting an unmet dental care need in 2015 than in 2012 (OR = 0.72, 95% CI 0.54, 0.95). Odds of low-income women having a dental care visit after Medicaid expansion were not significantly increased (OR = 1.05, 95% CI 0.80, 1.37).
Han, Ngyuyen, Drope, and Jemal (2015) [18] Medicaid expansion	The Medical Expenditure Panel Survey (MEPS), 2010–2012 Multivariate logistic, and two parts modeling approach with a logistic regression and Poisson regression	Low-income adults aged 18–64 (n = 9755 in Medicaid expanding states & 7455 in nonexpanding states)	Unmet dental care need Preventive dental service (dental checkup) Number of dental visits	The Medicaid non-expansion group was more likely to be unable to get or delay necessary dental care than the Medicaid expansion group, but the difference was not statistically significant (12.5% vs 14.0%, PR = 1.19, CI 0.98–1.43). The non-expansion group was less likely to have dental check-ups than the Medicaid expansion group (37.7% vs 42.7%, PR = 0.86, CI 0.79–0.94). The non-expansion group had 0.08 fewer dental visits annually than those in the Medicaid expansion group (CI 0.01–0.14).
Johansen and Richardson (2019) [19] ACA	The MEPS, 2012–2015 Analyses: adjusted Wald test	Adults aged 18 and older	The number of individuals per 1000 persons who had dental visits per month	The number of individuals who had received dental services in the past month increased in post ACA (67 persons per 1000 per month in 2012–2013 compared to 70 persons per 1000 per month in 2014–2015, *p* = 0.035).
Kotagal, Carle, Kessler, and Flum (2014) [20] ACA	The National Health Interview Survey (NHIS), 2009 and 2012 Difference-in-differences analysis	Adults aged 19–25 years and 26–34 years	Rates of inability to afford dental care in the past year	There was no significant difference in young adults’ (aged 19–25 years) reports of inability to afford dental care in the past year between 2009 and 2012 compared with adults aged 26–34 (DID = −2.6%, 95% CI, −5.61 to 0.61).
Lau, Adams, Park, Boscardin, and Irwin (2014) [21] ACA	The MEPS, 2009 and 2011 Logistic regression	Adults aged 18–25 (n = 3768 in 2009 & 3717 in 2011)	Rates of annual dental visit in past year	The rate of receiving an annual dental visit increased from 55.2% in 2009 to 60.9% in 2012 (*p* < 0.001). Adults aged 18–25 were more likely to receive an annual dental visit in 2012 compared with 2009 (OR = 1.3 CI, 1.1–1.4). After controlling for insurance types (full-year private; full year public; partial year uninsured; full-year uninsured), the degree of difference between 2009 and 2011 decreased, but remained significant (OR = 1.2, CI, 1.0–1.3, *p* < 0.05). After controlling for covariates, adults aged 18–25 were more likely to receive an annual dental visit in 2012 compared with in 2009 (OR = 1.3 CI, 1.1–1.5, *p* < 0.01).
Lyu, et al. (2020) [22] Medicaid expansion	The MEPS, 2011–2016 Difference-in-differences	Adults aged 19–64 whose newly eligible income level (below 138% FPL) for Medicaid	Preventive dental visit Dental treatments	Among states offering extensive dental benefits, the likelihood of preventive dental visits for Medicaid expansion states increased by five percentage points after implementation of Medicaid expansion (2014–2016) compared with non-Medicaid expansion states. Among states offering extensive dental benefits, the likelihood of dental treatments for Medicaid expansion states increased by four–five percentage points in 2014–2015, but the effect of Medicaid expansion decreased and became insignificant in 2016. Among states offering limited dental benefits, the likelihood of preventive dental visits for Medicaid expansion states increased by seven–eight percentage points after implementation of Medicaid expansion of the ACA (in 2014 and 2015) compared with non-Medicaid expansion states, but the effect of the Medicaid expansion decreased and became insignificant in 2016. Among states offering limited dental benefits, the likelihood of dental treatments for Medicaid expansion states increased by three–five percentage points after implementation of Medicaid expansion (2014–2016), but the increase was insignificant. Among states offering emergency only coverage, the likelihood of preventive dental visits for Medicaid expansion states increased by five–eight percentage points after implementation of Medicaid expansion compared with non-Medicaid expansion states, but increases were not significant. Among states offering emergency only coverage, the likelihood of dental treatments for Medicaid expansion states increased by five percentage points in 2015 and 2016 compared with non-Medicaid expansion states, but the increase in 2014 was not significant.
Peck, Sedgley, and Schwarz (2019) [23] ACA (dental benefits for Medicaid as part of Oregon Health Plan (OHP)	Electronic health records in the Oregon Health & Science University Graduate Endodontic Clinic (GES), 2010–2017 Chi-square	Patients (children 0–20 and adults 21–64) who attended in Graduate Endodontic Clinic	The number of non-surgical root canal therapy (NS-RCT)	There was significant increase in the number of NS-RCT provided for patients covered by OHP post-ACA compared to pre-ACA (152 in pre-ACA vs 674 in post-ACA, 363% increase), whereas there was a significant decrease in the number of NS-RCT provided for non-OHP patients (1032 in pre-ACA vs 844 in post-ACA) (*p* < 0.00001). The number of both of adults and children whose NS-RCT was covered by OHP increased their receipt of NS-RCT services in the post-ACA period, whereas the number of non-OHP adults and children decreased their receipt of NS-RCT (*p* < 0.00001).
Seo, et al. (2019) [24] ACA on access to community health centers	The Health Center Patient Survey, 2014 Logistic regression	Adults aged 18 or older who sought care at Community Health Center	Delayed or unable to get dental care need in the past 12 months	Those uninsured were more likely to have delayed or no dental care in the past 12 months than those with Medicaid (OR = 2.4, CI, 1.3–4.6).
Simon, Soni, and Cawley (2017) [25] ACA	The BRFSS, 2010, 2012, 2014, and 2015 Difference-in-differences analysis	Low-income (below 100% FPL) adults aged 19–64	Dental visit in the past year	There was no significant effect of Medicaid expansion on dental visits for low-income adults. Among childless adults, the probability of a dental visit increased by 4.1 percentage points post-ACA implementation, relative to pre-ACA passing (*p* < 0.01).
Singhal, Damiano, and Sabik (2017) [26] Medicaid expansion	The BRFSS, 2010 and 2014 Multivariate linear regression model	Low-income (annual household income $ <15,000) adults aged 21–64	Dental visit in the past 12 months	The probability of having had a dental visit in the past year among low-income residents of Medicaid expansion states with dental benefits decreased from 52.3% in 2010 to 50.4% in 2014 (*p* < 0.001). Among states that provided dental benefits, the probability of low-income parents visiting a dentist decreased from 56.0% in 2010 to 47.9% in 2014 in Medicaid expansion states (*p* < 0.0001). Among states that provided dental benefits, the probability of childless adults having had a dental visit in the past year increased from 48.6% in 2010 to 50.4% in 2014 in Medicaid expansion states (*p* < 0.0001).
Wehby, Lyu, and Shane (2019) [27] ACA on dental care use by dental benefits and dental supply (dentist availability in states)	The BRFSS, 2012, 2014 and 2016 Difference-in-differences	Low-income (<138% FPL) adults aged 18–64	Dental visit in the past 12 months	Low-income adults residing in Medicaid expansion states which offered extensive dental coverage were 5.8 percentage points more likely to have a dental visit in 2016 when compared to 2012 (*p* < 0.01). Low-income adults residing in Medicaid expansion states which offered limited dental benefits were 1.1 percentage points more likely to have a dental visit in 2016 when compared with 2012, but it was not significant. Low-income adults residing in Medicaid expansion states which offered emergency dental benefits were 7.1 percentage points less likely to have a dental visit in 2016 when compared with 2012 (*p* < 0.05). There was a significant effect of Medicaid expansion with extensive dental coverage in states with high numbers of available dentist or high dentist availability (the likelihood of dental visits increased by 6.7 percentage points in states with high dentist availability). There was no significant effect of Medicaid expansion providing limited dental coverage in areas with low or high concentrations of available dentists.
Zwetchkenbaum and Oh (2017) [28] Medicaid expansion	Medicaid enrollment & dental claims from Rhode Island Medicaid Management Information system, 2012–2015	Medicaid enrollees aged 18–64	Annual unduplicated numbers of dental care service use Percentage of Medicaid enrollees who had dental service	The number of Medicaid enrollees who received dental services increased by 46% between 2013 (33,800) and 2015 (49,312). However, the percentage of Medicaid enrollees who received dental services decreased from 39% in 2013 to 32% in 2015.
Nasseh and Vujicic (2017) [29] ACA	The Gallup-Healthways Wellbeing Index survey, 2010–2016 Difference-in-differences analysis of data	Adults aged 21–64 with household income at or below 138% FPL	Receiving a dental service in the last year	The younger age group increased by 0.39 percentage points in oral care use after implementing dependent expansion, relative to older age group (*p* < 0.05) Dental care use among low-income adults in expansion states with adult Medicaid dental benefits increased by 2.8% in 2015 (*p* = 0.049) and 2.8% in 2016 (*p* = 0.042), relative to states with non-Medicaid expansion states and without dental benefits. Dental care use among low-income adults in expansion states with adult Medicaid dental benefits increased by 2.6% in 2015 (*p* = 0.007) and 6.4% in 2016 (*p* < 0.001), relative to states with non-Medicaid expansion and with Medicaid-provided dental benefits to adults. Dental care use among low-income adults in expansion states with adult Medicaid dental benefits increased by 0.01% in 2015 (*p* = 0.995) and 5.8% 2016 (0.001), relative to states with Medicaid expansion and without Medicaid-provided dental benefits to adults.
Khoujs et al., (2020) [30] Medicaid expansion	The MEPS, 2011–2016	Dyads of children (6–18 years old) and parents (21–64 years old) in family below 125% FPL	Preventive dental service (cleaning, fluoride treatment or sealant application)	In states that covered preventive dental services for adults with Medicaid, the implementation of Medicaid expansion was not statistically significant with regard to the probability of children receiving preventive dental services (1.26 percentage points, CI: −3.74–6.27). In states that did not cover preventive dental services for adults with Medicaid, the implementation of the ACA was not followed by statistically significant changes in the probability of children receiving preventive dental services (3.3 percentage points, CI:−2.76–8.81).
Kino and Kawachi (2018) [31] ACA (Medicaid expansion)	The BRFSS, 2011–2016 Difference-in-differences analysis	Adults aged 18–64 years (n = 1,875,151)	Two indices of socioeconomic inequality in health service utilization (Having a dental visit in the past year): SII: Slope Index of Inequality RII: Relative Index of Inequality	Medicaid expansion was not associated with a reduction in income-based inequalities for having a dental visit in the past year.
Nasseh & Vujicic (2017) [32] ACA	The Gallup-Healthways Wellbeing Index survey, 2010–2014 Difference-in-differences analysis	Low-income (<138% FPL) adults aged 21–64	Having a dental visit in the past 12 months	Dental care use for low-income adults residing in Medicaid expansion states (providing dental benefits) increased by 2.9 percentage points (from pre- to post-ACA) compared with low-income adults residing in non-Medicaid expansion states without dental benefits. (*p* = 0.083). Dental care use for low-income adults residing in Medicaid expansion states (providing dental benefits) increased by 6.2 percentage points (from pre-to post-ACA) compared with low-income adults residing in non-Medicaid expansion states with dental benefits. (*p* = 0.043). Dental care use for low-income adults residing in Medicaid expansion states (providing dental benefits) increased by 1.2 percentage points (from pre- to post-ACA) compared with low-income adults residing in non-Medicaid expansion states with dental benefits. (*p* = 0.763).
Yoruk. (2018) [33] Dependent mandate	The MEPS, 2011–2013	Adults aged 23–29	Dental visits	The probability of dental care utilization decreased by 1.0–2.1 percentage points when young adults turned 26 and the number of dental office visit decreased at this cutoff age (from 0.014 to 0.030 times per month).
Soni. (2020) [34]	The BRFSS, 2010–2018 Difference-in-differences	Childless low-income adults aged 19–64 (below 100% FPL)	Visited a dentist in the past year	For low-income adults without children, ACA Medicaid expansion increased the probability of a dentist visit by 2.3 percentage points (*p* < 0.05).
Chalmers, Grover, and Compton (2016) [35] Medicaid expansion	The State Emergency Department Databases of the Healthcare Cost and Utilization (HCUP) in Kentucky, 2010–2014 Descriptive	Adults older than 21	ED discharges for conditions related to dental or oral health	The percentage of discharges for dental or oral health conditions by Medicaid increased (17.6% in 2013 to 49.7% in 2014). The percentage of discharges for dental or oral health conditions by the uninsured decreased (56.8% in 2013 to 20.5% in 2014). The percentage of those discharged with dental or oral health conditions among Medicaid enrollees increased in 2014 compared to 2013 (3.7% vs 2.7%).
Laniado, Badner, and Silver (2017) [36] ACA	The State Emergency Department Database for Minnesota, 2008 and 2014 Chi-square tests	Adult aged 18 and older	The total number of ED visits with non-traumatic dental conditions The rates of dental ED visits per 100,000 population and the number of dental ED visits (a percentage of total dental ED visits)	There was a 9.7% decrease in ED visits involving non-traumatic dental conditions between 2008 and 2014. The rate of ED visits involving non-traumatic dental conditions per 100,000 population decreased by 13.1% (612/100,000 in 2018 and 531/100,000 in 2014).
Rampa, et al. (2019) [37] ACA on ED use for periapical abscess	The Nationwide Emergency Department Sample (NEDS), 2008–2014 Chi-square	Patients who visited the ED with periapical abscess.	The rate of ED with periapical abscess visits per 100,000 people	The rate of ED visits with periapical abscess per 100,000 people increased from 151.31 in 2008 to 171.27 in 2014.
Elani et al. (2020) [38] Medicaid expansion	The state Emergency Department database (SEDD), 2012 & 2014	Low-income adults aged 19–64	ED visits with dental conditions	There was an overall increase in ED visits with dental conditions from 175,746 in 2012 to 200,198 in 2014. However, states that expanded Medicaid by offering adult dental benefits decreased ED visits for dental conditions (−14.1%).
Laniado, et al. (2020) [39] Medicaid expansion	The State Emergency Department Database for New York and New Jersey, 2010–2014		ED discharge with non-traumatic dental conditions	The percentage of ED discharges with non-traumatic dental conditions decreased in NY and NJ from 2010 to 2014. In NJ, ED discharges with non-traumatic dental conditions by uninsured patients decreased by 35 percent, whereas discharges with non-traumatic dental conditions by Medicaid patients increased by 57 percent. In NY, ED discharges with non-traumatic dental conditions decreased steadily from 2010 to 2014, whereas discharges with non-traumatic dental conditions by Medicaid patients remained relatively steady between 2010 and 2014.
Lee et al. (2020) * ACA	The MEPS, 2011–2016	Adults older than 18	Preventive oral checkup The number of dental treatment	There was an increase in preventive oral checkups received between pre-ACA and post-ACA, but the difference was not significant after controlling for education and poverty level. There was an increase in the number of dental treatments received between pre-ACA and post-ACA, but the difference was not significant after controlling for adults’ education and poverty level.

Notes. Abbreviations: ED: Emergency Department; MEPS: Medical Expenditure Panel Survey; ACA: Affordable Care Act; Study that is marked with an (*) is not discussed in this review.

## Supplementary Materials

The following are available online at https://www.mdpi.com/article/10.3390/ijerph18157865/s1, Supplementary for Methodology and Results. Figure S1: Forest Plot, Figure S2: Funnel Plot.
