# Supplementary material for: The Impact of the Affordable Care Act on Dental Care: An Integrative Literature Review
Source: Int J Environ Res Public Health. 2021 Jul 25;18(15):7865. doi: 10.3390/ijerph18157865 (PMC8345350; doi:10.3390/ijerph18157865)

Supplementary for Methodology and Results (Meta-analysis)

The Impact of the Affordable Care Act on Dental Care: An Integrative Literature Review

Jihee Song [1]*, Jeong Nam Kim [2]*, Scott Tomar [3] and Lauren N. Wong [4]

[1] Department of Family, Youth, and Community Sciences, University of Florida, Gainesville, FL 32611, USA; ssong@ufl.edu

[2] Department of Microbiology, College of Natural Science, Pusan National University, Busan 46241, Korea; kimjn@pusan.ac.kr

[3] Department of Pediatric Dentistry and Pediatric, University of Illinois Chicago, Chicago, IL 60612, USA; stomar@uic.edu

[4] School of Special Education, School Psychology, and Early Childhood Studies, University of Florida, Gainesville, FL 32611, USA; laurennwong@ufl.edu

* Correspondence: ssong@ufl.edu (J.S.); kimjn@pusan.ac.kr (J.N.K.)

To examine the strength of the evidence for dental care utilization, this review had the following research question for meta-analysis: Did enactment of the ACA lead to a statistically significant increase in dental care utilization?

Methodology

A random effects model using inverse variance weights was run to estimate the average effect size. ES were treated as dependent because several studies reported more than one outcome of interest. Though independent samples were the unit of analysis, dependence (e.g., multiple estimates from the same units) can still occur among studies, such as multiple experiments reported in a single study. [1] The random effects model was estimated by using robust variance estimation with the *robumeta* package in *R* (version 3.6.2). Robust variance estimation was chosen due to its ability to handle correlated data when the within-study covariance structure is unknown, it provides consistent estimates of the underlying population parameters (e.g., standard errors, point estimates, confidence intervals, and significance tests), and it can be adjusted for small sample sizes (e.g., when the number of studies is less than 40) [1]. The supplement provides methodology for effect size and heterogeneity.

Effect size

Effect sizes (ES) were calculated for dental care utilization between the pre- and post-ACA time periods. Of the 33 studies, we extracted data from 22 studies that included dental care utilization (having a dental visit or dental treatment) as outcomes. Of those 22 studies, nine were included in the analysis; 13 were excluded due to a lack of required information. For example, Yoruk [2] was excluded because the standard deviation of the dependent variable was not reported. Independent samples were used as the unit of analysis to address ES dependency issues. ES were calculated by using (a) frequency counts and sample sizes; (b) means, standard deviations (SD), and sample sizes; and (c) odds ratios and sample sizes. The Campbell Collaboration online effect size calculator [3] was used to calculate probit *d* by using frequency counts and sample sizes and to calculate Cohen's *d* by using means, SDs, and sample sizes for pre- and post-ACA dental care utilization measure. Cohen's *d* was calculated as the difference between treatment and control groups' post-ACA measures after adjustments for pre-ACA measures and other covariates, divided by the pooled SD from pre-implementation measures. For samples that only reported post-ACA measures, SD, and sample sizes, unadjusted ES were calculated, which do not take into account other variables that might have had an influence on the outcomes. Odds ratios and sample sizes were extracted, converted to Pearson's *r*, and then converted to Cohen's *d*. The variance of Pearson's *r* was obtained by using Campbell Collaboration online calculator and the converted odds ratio and sample size. Because this yielded a variance of zero, these variances were Winsorized, in which they were

recoded back to the next highest variance to reduce the effect of possibly spurious outliers [4]. For studies that included more than one outcome, separate ES were calculated for each outcome.

Heterogeneity and publication bias

Heterogeneity was assessed by using the I^2 statistic, in which the proportion of observed variance that reflects the real difference in effect sizes is reflected [5]. An I^2 greater than 75% was considered to indicate considerable heterogeneity, where a high proportion of observed variance is explainable [6]. Publication bias was assessed by using a funnel plot.

Results

Results from the nine studies included in the meta-analysis indicated a small, non-significant, and positive effect (d = 0.293, SE = 0.292, p = 0.346), where an increase in dental care utilization was observed but this increase was not statistically significant. A forest plot is presented in figure S1. Though the overall effect size was not significant, a large proportion of observed variance indicates a real difference in ES (I^2 = 99.98%). To examine whether type of outcome (e.g., dental visit in the past year or number of dental services) could account for some of the variance, we conducted a subgroup analysis. However, there were low degrees of freedom (i.g., fewer than four) for the different outcomes that led to uninterpretable results. Also, based on the asymmetry of the funnel plot (figure S2), there is evidence of publication bias. However, the exclusion of a large number of studies due to a lack of reported information presents a reasonable explanation for the asymmetry of the funnel plot.

References

1. Fisher, Z.; Tipton, E. Robumeta: An R-package for robust variance estimation in meta-analysis. 2015.
2. Yörük, B.K.. Health insurance coverage and health care utilization: Evidence from the Affordable Care Act's dependent coverage mandate. *Forum Heal. Econ. Policy*. 2018, 21, 2, 1–24. doi: 10.1515/fhep-2017-0032.
3. Wilson, D.B. Practical Meta-Analysis Effect Size Calculator [Online calculator] Available on online: https://www.campbellcollaboration.org/research-resources/effect-size-calculator.html. (Accessed: 16-Feb-2021).
4. Lipsey M.W.; Wilson, D.B. *Practical meta-analysis*. SAGE Publications Inc., 2001.
5. Borenstein, M.; Hedges, L.V.; Higgins, J.P.T.; Rothstein, H.R. *Introduction to Meta-Analysis*. Chichester, UK: John Wiley & Sons, Ltd, 2009.
6. Cooper, H. *Research Synthesis and Meta-Analysis: a step-by-step approach*. SAGE Publications Inc., 2017.

Figure S1. Forest Plot

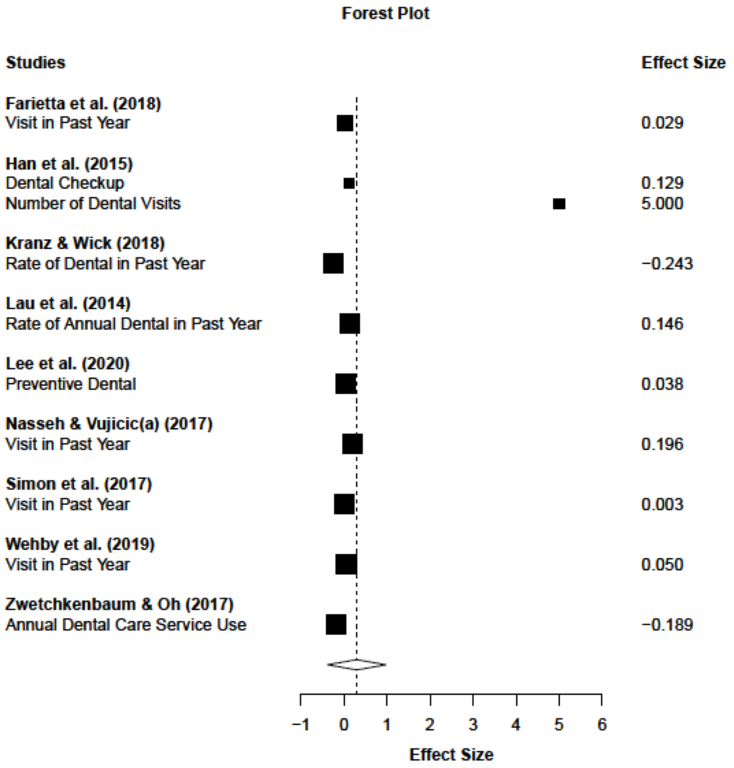

Figure S2. Funnel Plot

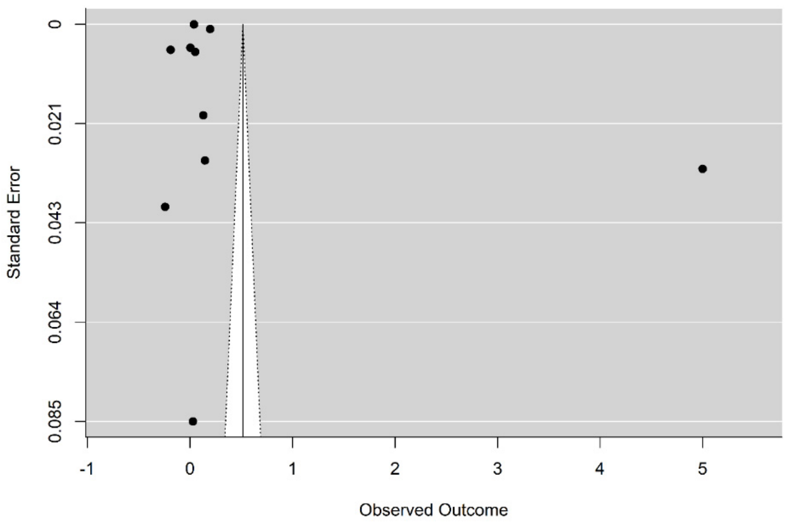

Supplement: Supplementary file 1 [file ijerph-18-07865-s001.zip › ijerph-1277032-supplementary.pdf]
